# Complete Genome Sequences of Seven Avibacterium paragallinarum Isolates from Poultry Farms in Pennsylvania, USA

**DOI:** 10.1128/MRA.00654-20

**Published:** 2020-07-02

**Authors:** Maurice Byukusenge, Ruth H. Nissly, Lingling Li, Traci Pierre, Tammy Mathews, Eva Wallner-Pendleton, Patricia Dunn, Denise Barnhart, Sean Loughrey, Sherrill Davison, Donna J. Kelly, Deepanker Tewari, Bhushan M. Jayarao, Suresh V. Kuchipudi

**Affiliations:** a Animal Diagnostic Laboratory, Pennsylvania State University, University Park, Pennsylvania, USA; b Pennsylvania Animal Diagnostic Laboratory, New Bolton Center, University of Pennsylvania, Kenneth Square, Pennsylvania, USA; c Pennsylvania Veterinary Laboratory, Harrisburg, Pennsylvania, USA; d Center for Infectious Disease Dynamics, Pennsylvania State University, University Park, Pennsylvania, USA; Broad Institute

## Abstract

Avibacterium paragallinarum, the causative agent of infectious coryza, causes significant economic losses to the poultry industry due to increased culling rates in growing chickens and decreased egg production in layers. We present the complete genome sequences of seven strains of Avibacterium paragallinarum isolated from poultry farms in Pennsylvania during 2019.

## ANNOUNCEMENT

Avibacterium paragallinarum, formerly classified as Haemophilus paragallinarum (1), causes infectious coryza (IC) in poultry. IC is a highly contagious respiratory disease of chickens resulting in high mortality, reduced egg production, and huge economic losses to the poultry industry worldwide (2–4). Since early 2019, there have been several outbreaks of IC in Pennsylvania. The complete genome sequences of seven Avibacterium paragallinarum isolates from these outbreaks were deposited in GenBank. Currently, there are very few whole-genome sequences of *A. paragallinarum* in public databases, and these genome sequences will facilitate further molecular epidemiologic analyses.

Samples submitted to the Pennsylvania Animal Diagnostic Laboratory System (PADLS) from IC-suspected chickens were streaked onto chocolate agar and incubated for 24 h at 37°C with 5% CO_2_ (5). Isolated single colonies were grown overnight in brain heart infusion broth (BD) supplemented with chicken serum and NAD. Bacterial DNA was extracted using the Qiagen Genomic-tip 100/G following the manufacturer’s instructions. For each isolate, two sequencing platforms, MinION from Oxford Nanopore Technologies (ONT) and Illumina MiniSeq, were used to leverage the accuracy of the short reads from Illumina and the long reads from ONT. The Illumina Nextera DNA Flex library prep kit and 1D native barcoding genomic DNA protocol (EXP-NBD104 and SQK-LSK109; Oxford Nanopore Technologies) were used to prepare the library for Illumina and MinION sequencing, respectively. The quality of the Illumina short reads (150 bp) was assessed using FastQC version 0.11.9 (6), and no further quality control was required. The ONT reads were base called using Guppy version 3.1.5 (available on the ONT community website). The program was run in “fast” mode with the option to simultaneously demultiplex the reads with barcode sequences. Filtlong version 0.2.0 (7) was used for quality control of the ONT reads. The options were set to filter out the smaller reads and trim off the regions with the lowest quality scores. This resulted in a total of 350 Mbp (coverage, ∼145×) of the longest reads with the highest quality scores.

Unicycler version 0.4.8 (8), with default options, was used to perform *de novo* hybrid assembly. For each genome, the assembly resulted in a single circular contig, which was rotated to allow all genomes to start at the same site (the *DnaA* gene). The assembled genomes were submitted to GenBank and were annotated using the NCBI Prokaryotic Genome Annotation Pipeline (PGAP) (9). These isolates were previously identified by matrix-assisted laser desorption ionization–time of flight (MALDI-TOF) mass spectrometry and were later identified by *in silico* species identification using KmerFinder (10). FastANI (11) was used to calculate the average nucleotide identity (ANI) values between the genome sequences. The ANI values between all of the genomes reported here are above 99.99% and are closer to those of two genomes from Peru, strains 72 (ANI, 99.86%) and FARPER-174 (ANI, 99.74%) (12, 13), and strain AVPG2015 from Mexico (ANI, 99.88%).

### Data availability.

These data were deposited in the NCBI GenBank database under the BioProject accession no. PRJNA625662. The complete sequences and their corresponding raw reads have been deposited in GenBank and the SRA, and the details are provided in Table 1.

**TABLE 1:** Metrics and accession numbers of genome sequences of Avibacterium paragallinarum isolates from Pennsylvania

Isolate	Genome size (bp)	GC content (%)	Total no. of genes	Illumina data:			Oxford Nanopore data:			SRA accession no.	GenBank accession no.
				Total no. of reads (bp)	Avg read length (bp)	Avg coverage (×)	Total no. of reads	*N*_50_ (bp)	Avg coverage (×)		
ADL-AP01	2,415,542	40.91	2,330	821,856	148	50	126,648	14,949	400	SRS6501300	CP051642
ADL-AP02	2,416,187	40.92	2,328	988,642	148	61	143,612	14,599	443	SRS6501303	CP051641
ADL-AP07	2,415,993	40.91	2,230	1,034,010	148	63	203,889	13,633	599	SRS6501304	CP051640
ADL-AP10	2,415,552	40.91	2,334	554,656	148	34	228,213	13,294	523	SRS6501305	CP051639
ADL-AP15	2,415,950	40.92	2,337	697,442	148	43	155,612	13,500	450	SRS6501306	CP051638
ADL-AP16	2,415,855	40.91	2,331	2,170,560	147	132	281,174	13,210	610	SRS6501301	CP051637
ADL-AP17	2,415,699	40.91	2,331	2,671,088	148	164	115,998	15,175	378	SRS6501302	CP051636
